# Tough, Tougher, Toughest

**Published:** 1872-04

**Authors:** 


### TOUGH, TOUGHER, TOUGHEST!

BY DR. NED.

We have heard so much of the entire helplessness of the human race, of the extreme tenderness of infancy, and the infirmities of the flesh, that most people have been led to conclude that we cannot endure anything; but I am satisfied that this is all nonsense, and furthermore, that of all living creatures, the human being is by far the toughest in every sense of the word. The mental effort of an earnest student demands more physical force every day, than is expended by many powerful animals in a month. I have known men who consumed tobacco and whisky enough from morning until night, to make a score of elephants crazy, and depopulate a good-sized neighborhood of its dogs and cats in a few hours; (who don't wish that such persons would do themselves a favor, at the same time rid the community of useless curs and our back yards of the feline swarms, so that people could rest in peace?) but they *will* insist upon squirting the filthy juice on the street and in the drawing-room, while the turbulent kittens make night hideous, and independent curs, by dozens, tumble pell-mell over the fences, doing their best to make every horse they see run away and kill his driver! Oh, what a waste of tobacco! What a pity such brutes are allowed to go without it!

It is not my intention to write an essay upon whisky and tobacco. I will only remark that no animal except man, is fool enough to drink the liquid fire, no other is mean enough to desire it, or so debased as to get on "*benders*," and it is doubtful if any other would be tough enough to stand them.

As for tobacco, it is very difficult to acquire the taste for it, and mankind, in all probability, would never have become addicted to its use, had it not been that to the Lords of Creation alone was given the *sublime* faculty of spitting; cows, horses, pigs, dogs, and all of this low order of creation, that go on all-fours, with their mouths hanging down, looking as though they could spit just as easy as not, seem to be entirely too neat in their habits of life to indulge in the luxury. The man, walking erect, looking upwards, as if in search of a more exalted destiny; well dressed, with yellow kids, a gold-headed cane, tall hat, clean bosom, and stiff collar, stops on the street, halts in the dance, wipes his eyes at a funeral, and bends over to squirt tobacco juice through his moustache, without touching a hair, and looks ridiculous. Every day he uses enough of the filthy weed to kill a score of strong men, who have not acquired the villainous practice, wastes time enough spitting, wiping, and hunting a good place to spit, to make him rich at the age of sixty.—What a fool he is, and how very tough he must be.

After all, whisky and tobacco are good in their places, and I don't know how the criminal courts could be sustained, the jails kept full, and the hungry scaffolds fed, or the devil drive much of a business, if he had no tobacco to destroy stomachs with and make people spit themselves dry as graven images, with whisky to touch off the human tinder, and so start little bits of hells everywhere; he knows his business, attends to it well, and greets his subjects with demoniac grace and subtle laughter when they lie, cheat, rob, swear, murder, spit and drink!

Mr. Editor, if *your* devil will pardon this digression, I will risk the other one without any apology, and say no more of whisky and tobacco this time.

A man can travel further in a month or year than any horse, and he must be tough, for he can endure more suffering, privation and hunger, than any animal under the sun, and live. Some animals will lose their master, sit down in sorrow alone, and die; but men will bury the partner of their lives, cry at the grave, weep all the way home, put on black clothes, and marry again in six weeks, and be as happy as ever. Therefore, it is easy enough to be seen, that we are too tough to die of sorrow. Intellectually, morally, physically and socially, man is tough. In every sense of the word he has a tough time of it, for it is tough to live right, tough *not* to live right, and worse still, it is fearfully tough not to die right. Yet, a large proportion of the race do everything wrong, and at last they die wrong, and if they can stand *that*, well, no one will deny that they are tough enough!

Although men endure almost everything and live, they are not as tough by any means, as the women. It would kill a man in a very short time, to fix ten or a dozen pounds of hair upon the back part of his head, cut out the upper part of his coat, vest and underclothes, so as to expose to the air and view as much of the chest as possible and avoid the civil law; then put on him a corset filled with stays, and girded up well so as to compress the stomach and bowels in about one-half of their natural dimensions; then swallow turkey, beef, salad, coffee, ice-cream and pastry without stint, and hang to his hips from eighty to one hundred yards of goods, (weighing from eleven to-twenty-five pounds), the lower six or seven yards trailing behind him, sweeping up the dust and dirt of streets or carpets—while every few minutes some fellow stands on the nuisance, almost jerking him down—and then to finish him up in full fashion, put on his feet shoes as thin as paper, with heels so elevated as to oblige him to walk on tiptoe; now, how long do you think, dear reader, a man so fixed, could survive? You may have heard of the silly chap who attempted the trial of all these new styles to test his powers of endurance, and who in six hours complained of great heat in the head, with all the symptoms of brain fever; in twelve hours had an attack of pleurisy, from the nakedness of his chest, the best medical minds declaring that he was a candidate for consumption. In less than six hours more, from the compression by his corset, and the extra set of ribs going the wrong way, supplying the abdomen with the article forgotten by nature, he shared all the signs of inflammation of the stomach and bowels; was obliged to have the incubus removed from his hips, and the dirt collected by his automatic broom (or trail) cleaned from his body so fomentations, or a blister, could get down to the skin; but all to no purpose, for the poor fellow had only a few more hours to live. While he was cantering on tip-toe, some person, who was pressed to the last extremity, for an inch of standing room, unfortunately put his foot upon the intolerable nuisance, which so provoked the indignation of the already sick, tired, and over-freighted wearer, that he actually forgot his obligations, like Peter, and swore; and worse still, he struck the innocent and unsuspecting offender. A row ensued, the police lodged both parties in jail; medical skill was of no avail, the next day he wound up his earthly career, and the honest parson, with a few heart-broken and bereaved relatives and friends, consigned him to the tomb's cold, cheerless, and silent embrace; stifling for a moment the hot tear, and stilling the troubled heart with the glorious consolation, "The Lord giveth and the Lord taketh away." Poor unfortunate, sleep in peace, let no harsh tongue ever tell to those sad ones who mourn your loss, that you got sick, backslid, and died in two days, because you tried the fashions for *fun*, for a few hours, which women practice and seem to enjoy, for years! The sudden demise of this tough man, if true, demonstrates that women are tougher: but the pallid cheek, the blanched lip, the deep furrows ploughed in the face of youth, the bended form, the hurried respiration, the faint, shrill, feeble voice, the husky throat and hacking cough, are but the flags of death, hanging over the forms of beauty. Woman, it is only a question with you, for insulted, outraged and violated nature, will find a day of certain retribution, and your offspring, enervated by your follies and ruined by your practices, could justly inscribe upon your tomb: "A fashionable suicide."

As tough as women may be, they cannot stand as much as babies, and I doubt if any animal in the world could live very long and tolerate the treatment to which the loving mother and the careful nurse subjects the baby. If babies were not the toughest of all things, the human race would be extinct in a single generation. Imagine a strong man or woman, put over the road traveled by a baby, in a "well fixed" family; first, we will have nurse ready, and strong enough to handle a grown person with ease, *grab him* the moment he is ushered into the *polar regions* from the *torrid* zone, and after fifteen minutes, places around his body snugly, so as to prevent the expansion of his chest, a few thicknesses of flannel, which will also serve to keep the ribs from flying away from the body, to which strips are attached, several yards of cloth, extending so far beneath the feet as to entirely prevent the motion of the extremities; now, he can scarcely breathe, and to enjoy the luxury of kicking, at such a moment, is out of the question: the dressing is finished substantially, by putting over the whole, a very nice, warm, pair of armholes, well secured to a few more yards of cambric, and all extending two or three times the length of the body, beyond the feet, where there is nothing to be kept warm. The arms, neck, and chest, find no protection whatever except from the comfortable holes already mentioned; but the toilet is completed, and the nurse stands ready with a spoon and a dish of milk, or panada, a paper of magnesia, a bottle of castor-oil, and a full supply of Mrs. Winslow's Magic Soother. Nature, I have observed, makes calculations for birds, fishes, brutes and all such *important beings*, but forgot babies. I suppose the neglect was owing to the press of business on hand the day the bill of fare was made out, or it may be, that the first pair, starting out as they did, of age, and full fledged, no calculations were deemed important for little folks; or, it may be that there were no babies contemplated in the original plan; but I will not attempt to unravel the mystery; nevertheless, there is a "screw loose" somewhere, and fortunately the nurse, with her cap-border standing at an angle of ninety degrees, knows exactly what to do, and at once begins to stuff the child as deliberately as though she was making sausage for a hotel. She is delighted to see the new boy swallow, and that it *must* do or choke, for its mouth is full, the problem being swallow or die. After a while, of course, it gets brim full, and begins to run over at the mouth, in a stream; the nurse sees it and is as expert with her spoon as the Spaniard with his lasso, for she will catch the end of the stream going a three minute gait, double it up, and tuck it back in the mouth as quick as a flash. Now, the stuffing over, the packing begins, and the poor thing, filled as full as a tick, begins to cry from the agony of distention. Well, it must be trotted now, the faster and the harder the better. It weeps more, it must be rolled; it is in agony, she puts it upon its stomach, doubles the trotting gait, and keeps time on its back, sometimes with one hand, oftener with both, reminding one of the double strokes in churning. The effects of this operation are soon manifested, for the sufferer vomits, and the nurse exclaims, "no wonder, for the stomach is sour, and the milk has curdled." Of course it has, for it has been churned long enough! Well, she corrects the acidity with magnesia, makes it drunk with the soother, and now, all sensation gone, she is master of the situation. So she stuffs and packs the infant once more, and lays it away for a few hours to be aroused for a nice little dose of caster oil, which it seems to enjoy very much indeed, for the nurse gently holds fast to its proboscis to keep it from the spoon, so eager is the child to get at it. So, day after day the poor thing is stuffed, crammed, and packed, trotted, rolled and rocked, made drunk, doctored and physicked. Generally they die, as is seen by the small graves; now and then, one lives. But if they were not exceedingly tough, the mothers and nurses would make a clean sweep of the Nursery. If man was an adjective, instead of a noun, we would class him as follows: Positive man, comparative woman, superlative baby.

---

THE Jacksonville (Florida) *Union* gets off this religious joke on one of its colored brethren who attempts to pray. The good colored deacon was praying for the recovery of a sick sister, and ended this way : "Oh ! Lord, help her. O ! Lord, make her well. Oh ! Lord, if you can't make her well, then, oh ! Lord, help her to grin and bear it !"

---

A YOUNG MAN in Hartford read somewhere that more deaths occurred at five o'clock in the morning than at any other hour, and now gets up regularly at four, in order to be out when Death makes his morning calls.

